# Precision Behavioral Management (PBM) and Cognitive Control as a Potential Therapeutic and Prophylactic Modality for Reward Deficiency Syndrome (RDS): Is There Enough Evidence?

**DOI:** 10.3390/ijerph19116395

**Published:** 2022-05-24

**Authors:** Margaret A. Madigan, Ashim Gupta, Abdalla Bowirrat, David Baron, Rajendra D. Badgaiyan, Igor Elman, Catherine A. Dennen, Eric R. Braverman, Mark S. Gold, Kenneth Blum

**Affiliations:** 1 The Kenneth Blum Behavioral & Neurogenetic Institute, LLC., Austin, TX 78701, USA; margbetts@hotmail.com (M.A.M.); catherine.a.dennen@gmail.com (C.A.D.); pathmedical@gmail.com (E.R.B.); 2 Future Biologics, Lawrenceville, GA 30043, USA; ashim6786@gmail.com; 3 Department of Molecular Biology, Adelson School of Medicine, Ariel University, Ariel 40700, Israel; bowirrat@gmail.com; 4 Center for Psychiatry, Medicine, & Primary Care (Office of Provost), Division of Addiction Research & Education, Western University Health Sciences, Pomona, CA 91766, USA; dbaron@westernu.edu; 5 Department of Psychiatry, South Texas Veteran Health Care System, Audie L. Murphy Memorial VA Hospital, Long School of Medicine, University of Texas Medical Center, San Antonio, TX 78229, USA; badgaiyan@gmail.com; 6 Center for Pain and the Brain (P.A.I.N Group), Department of Anesthesiology, Critical Care & Pain Medicine, Boston Children’s Hospital, Harvard Medical School, Boston, MA 02115, USA; dr.igorelman@gmail.com; 7 Department of Psychiatry, Washington University School of Medicine, St. Louis, MO 63110, USA; drmarkgold@gmail.com; 8 Institute of Psychology, ELTE Eötvös Loránd University, Egyetem tér 1-3, 1053 Budapest, Hungary; 9 Department of Psychiatry, School of Medicine, University of Vermont, Burlington, VT 05405, USA; 10 Department of Psychiatry, Wright State University Boonshoft School of Medicine, Dayton VA Medical Centre, Dayton, OH 45324, USA

**Keywords:** dopamine, hypodopaminergia, Genetic Addiction Risk Severity (GARS) test, pro-dopamine regulation (KB220), Restoregen ®

## Abstract

This brief commentary aims to provide an overview of the available and relatively new precision management of reward deficiencies manifested as substance and behavioral disorders. Current and future advances, concepts, and the substantial evidential basis of this potential therapeutic and prophylactic treatment modality are presented. Precision Behavioral Management (PBM), conceptualized initially as Precision Addiction Management (PAM), certainly deserves consideration as an important modality for the treatment of impaired cognitive control in reward processing as manifested in people with neurobiologically expressed Reward Deficiency Syndrome (RDS).

## 1. Precision Behavioral Management

**Figure 1:**
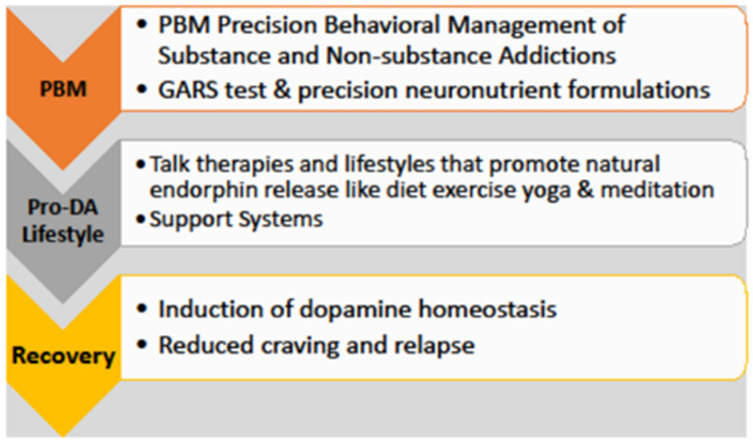
Precision Behavioral Management (PBM) platform. Reprinted/adapted with permission from Ref. [127]. Gold et al. copyright 2021.

**Figure 2:**
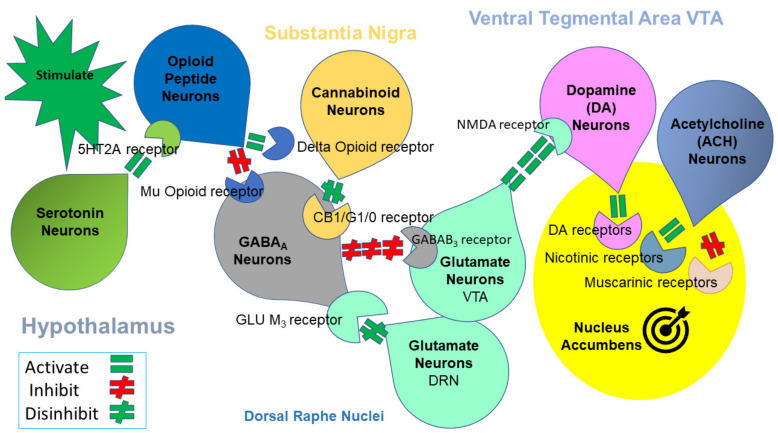
Mesolimbic Brain Reward Cascade [128]. This cartoon illustrates the interaction of the known major neurotransmitter pathways involved in the Brain Reward Cascade (BRC). In the hypothalamus, environmental stimulation results in the release of serotonin, which in turn, via, for example, 5HT-2a receptors, activates (green equal sign) the subsequent release of opioid peptides from opioid peptide neurons. Then, Substantia Nigra, the opioid peptides move to possibly two different opioid receptors with different effects. One inhibits (red hash sign) through the mu-opioid receptor (possibly via enkephalin) to GABA_A_ neurons. Another stimulates (green equal sign) cannabinoid neurons (the Anandamide and 2-archydonoglcerol, for example) through beta-endorphin-linked delta receptors, which inhibit GABA_A_ neurons. In addition, when activated, cannabinoids, primarily 2-archydonoglerol, can indirectly disinhibit (green hash sign) GABA_A_ neurons through the activation of G1/0 coupled to CB1 receptors. In the Dorsal Raphe Nuclei (DRN), glutamate neurons can then indirectly disinhibit GABA_A_ neurons in the Substantia Nigra through activation of GLU M_3_ receptors (green hash sign). GABA_A_ neurons, when disinhibited, will, in turn, powerfully (red hash signs) inhibit VTA glutaminergic drive via GABAB 3 receptors. At the Nucleus, Accumbens ACH neurons may stimulate both muscarinic (red hash) and nicotinic (green hash). Finally, glutamate neurons in the VTA will project to dopamine neurons through NMDA receptors (green equal sign) to preferentially release dopamine at the Nucleus Accumbens (NAc), shown as a bullseye, indicating a euphoria, or “wanting” response. The result is dopamine release; low release is (endorphin deficiency), and unhappiness is felt. General (healthy) happiness depends on the dopamine homeostatic tonic set point (with permission) [22]. Notably, various hypotheses have explained the findings that led to the modern known correlates of neurotransmitter interactions within this brain reward circuitry.

The Precision Behavioral Management (PBM) platform is related to addiction medicine, with the first USA and foreign patents related to the accurate Genetic Addiction Risk Severity (GARS^®^) test. Blum and Noble (JAMA, 1990) [1] found the first confirmed association of the DRD2 gene A1 allele with severe alcoholism and other Reward Deficiency Syndrome (RDS) behaviors, and Blum et al. have developed the GARS test and the pro- dopamine regulator, a nutraceutical neuronutrient (Research ID Code KB220) [2,3,4,5,6,7,8,9,10,11,12,13,14,15,16,17,18,19,20,21,22,23,24,25,26,27,28,29,30,31,32,33,34,35,36,37,38,39,40,41,42,43,44,45,46,47,48,49,50,51,52,53,54,55,56,57,58,59,60,61,62,63,64,65,66,67,68,69,70,71,72,73,74,75,76,77,78,79,80,81,82,83,84,85,86,87,88,89,90,91,92,93,94,95,96,97,98,99,100,101,102]. The basis of these addiction treatment interventions is research that identified a neurotransmitter network function within the Mesolimbic and Pre-Frontal Cortex (PFC) brain regions that regulates the final reward and motivational pathway of “wanting,” causing neuronal dopamine release (see Figure 1 and Figure 2) [103,104,105,106,107,108,109,110,111,112,113,114,115,116,117,118,119,120,121,122,123,124,125,126].

## 2. The Brain Reward Cascade

The cascading interaction of neurotransmitters and second messengers results in the correct release of dopamine within the NAc and across many brain regions. These regions are involved in motivation, cognition (memory), pleasure, stress reduction, drug reinstatement, decision making, recall, wellbeing, and cravings. The result is to provide *homo sapiens* with a usual happiness setpoint (i.e., dopamine homeostasis) [126], reflected in resting-state functional connectivity (rsFC) in neuroimaging studies [127].

In the neurophysiologic reward system, repeated frequent acute dopamine stimulation becomes chronic stimulation and leads to a dysfunctional hypodopaminergic state, rendering the reward system less responsive to natural reinforcers, a symptom of RDS [126,128]. The stimulation can be from euphorigenic substances, non-substances like gambling, or severe stressors like pain and anxiety. Chronic stimulation causes dopamine depletion (Hypodopaminergia). Reward deficiency results from depleted or hereditary hypodopaminergia, potentially reflected in a host of personality traits and mental and medical disorders that have been associated in genetic studies with dopamine depleting alleles. These symptoms and disorders create the diagnostic criteria for RDS and include, but are not limited to, novelty seeking, schizophrenia, obesity, chronic pain, post-traumatic stress disorder (PTSD), major depression, and attention deficit hyperactivity disorder (see Table 1) [129].

As alluded to above, reward deficiencies may also occur in the absence of dopaminergic stimulation by exogenous factors due to specific polymorphic alleles that alter the function of genes in the reward cascade. One example is the A1 allele of the D2 dopamine receptor gene that causes a reduced number of dopamine receptors in the mesolimbic NAc. An essential feature of RDS is the lack of integration between cognition, perception, and emotions occurring due to (1) substantial dopaminergic surges in reward, motivation, and learning centers leading to neuroplasticity in the striato-thalamic-frontal cortical loop, with ensuing top-down dissociation from the subcortical activity; (2) hypo-functionality of the excitatory glutamatergic afferents from the amygdala–hippocampus complex failing to produce bottom-up restraint of the striato-thalamic-frontal cortical loop [130,131,132,133,134].

The above aberrations may be a target of neuromodulation with therapeutic interventions and prophylaxis of addictive and related disorders. PBM combines the GARS test that identifies RDS risk polymorphisms and is used to ascertain the neuropathways involved in the tested individual’s hypodopaminergic risk neurotransmission finite pathways. These pathways are used in an algorithm to select a neuronutrient formulation of KB220 for that individual to induce the desired dopamine homeostasis by balancing genetic and epigenetic (neuro-molecular) brain reward activity [134].

## 3. Genetic Addiction Risk Severity (GARS)

The GARS test uses saliva samples and polymerase chain reaction (PCR) to identify dysfunctional polymorphisms of reward genes [135]. Genes express proteins that determine neurotransmitter function [136]. People who have Single Nucleotide Polymorphisms (SNPs) of genes in their DNA that cause dysfunctional dopaminergic neurotransmission are at risk of RDS behaviors, including addictions. The development of the GARS test used the reward cascade of neurotransmission to identify eleven SNPs that cause hypodopaminergia from ten reward genes [137,138,139,140,141,142,143].

Hypo-dopaminergia refers to a reduced dopamine function in the brain reward circuitry. As stated in the paper and indicated by the interrelatedness of at least seven main neurotransmitter pathways involving synthesis, vesicular pre-neuronal storage, mitochondrial catabolism, synaptic catabolism, neuronal clearance via transporters, receptor affinity, and number, carrying any one of the eleven polymorphic alleles could explain low dopamine function (see Table 1). One example is the finding by Noble and Blum that a progressively reduced Bmax was found in subjects with A2/A2, A1/A2, and A1/A1 alleles of the DRD2 gene, with subjects with A2/A2 having the highest and subjects with A1/A1, the lowest mean values (see Table 2).

The GARS test examines the sum of many related polymorphisms instead of one gene alone to predict genetic risk for RDS behaviors. It is possible to find a significant association with the degree of risk for all addictive behaviors (RDS). In conjunction with the Institute of Behavioral Genetics at Colorado University, unpublished research compared the GARS test with the Addiction Severity Index (ASI) in 273 subjects from substance treatment centers to determine drug and alcohol risk severity. Alcohol risk severity *p* < 0.04 predicted with seven or more alleles, and drug risk severity *p* < 0.05 predicted with four or more alleles. Understanding the genetic antecedents to Alcohol Use Disorder (AUD) may help explain potential neuroplasticity in those dependent on alcohol or other substances of abuse [142].

## 4. Precision Behavioral Management (PBM) System

The PBM system uses the patented, commercially available GARS test with a nutraceutical Precision Neuronutrient-Research ID code: KB220 [143,144,145,146].

The GARS test identifies RDS risks and determines the neuropathways involved in hypodopaminergic risk to identify the neuronutrient formulation of KB220 via algorithm and create balanced genetic and epigenetic (neuro-molecular) brain function, the desired induction of dopamine homeostasis.

## 5. Cognitive Control of Reward Processing

This Special Issue is not only about risky behaviors (addiction) but also cognitive control of reward processing; here, we provide a few examples, emphasizing the role of dopamine in addiction and cognition. The neuronal release of dopamine in the limbic system relies on many neurotransmitters, peptides, and second messengers to impact the dopamine released at the NAc, which is critical to feeling good [127].

There are many examples of cognitive control of various aspects of drug and non-drug addictive behaviors. For example, the ability to resist the urge to eat is in part a function of the homeostatic functioning of neuronal circuits involved in top-down control to oppose the conditioned responses that predict reward from eating the food and the desire to eat the food. Moreover, imaging studies by Volkow’s group of this non-drug behavior have revealed that obese probands have impairments in dopaminergic pathways that regulate neuronal systems linked with reward sensitivity, conditioning, and control [147]. There is evidence that neuropeptides regulate energy balance (homeostatic processes) via the hypothalamus and subsequently modulate the activity of dopamine cells and their projections into the regions involved in the rewarding processes that modulate food intake.

Substance use disorders (SUDs), while effecting billions worldwide, are indeed preventable. Thousands of articles attest to the well-known effects of substance misuse that cause many physiological, molecular, and cellular changes in specific brain regions. Moreover, these neuroplastic changes have a role in seeking behaviors seen in substance and non-substance addictions [148,149].

Notably, many studies have focused on the dopamine neurons of the ventral tegmental area (VTA) and the regions where these neurons terminate: the striatum, the prefrontal cortex [148], and the amygdala. Specifically, decreases in dopamine receptors and transmission have been found in chronic users of psychostimulants [150], cannabis [151], opioids [152], alcohol [153], and nicotine [154].

Another example is the role of cognitive control and reward processing in Internet Gaming Disorder (IGD). There are indeed common neurochemical mechanisms that have been observed with both IGD and SUD [155]. Specifically, Functional Magnetic Resonance Imaging (fMRI) investigations of the resting state and measures of gray matter volume have shown that Internet game playing is associated with changes to brain regions responsible for attention and control, impulse control, motor function, emotional regulation, sensory-motor coordination [156], and stress processing [157]. Most interestingly, IGD was associated with reduced white matter density in brain regions involved in decision making, behavioral inhibition, and emotional regulation [158]. Playing videogames is also associated with changes in reward inhibitory mechanisms and loss of control [159]. In addition, Tain et al. [160] reported that D2 receptor activity is significantly associated with glucose metabolism in subjects with IGD, suggesting that D2/5-HT2A receptor-mediated dysregulation of the orbitofrontal cortex could underlie a mechanism for loss of control and compulsive behavior in IGD individuals.

There is also evidence from experiments that used lentivirus tools for over-expression or silencing of the dopamine transporter (DAT) gene in animals. Behavioral profiles that evaluated motivation and self-control compared to controls revealed significant differences. Specifically, DAT over-expressing rats showed increased impulsivity. The authors concluded that an attenuated dopaminergic tone following altered accumbal DAT function may subserve a sensation-seeker phenotype with vulnerability to lack of impulse control [161,162,163].

## 6. Summary

In summary [2,3,4,5,6,7,8,9,10,11,12,13,14,15,16,17,18,19,20,21,22,23,24,25,26,27,28,29,30,31,32,33,34,35,36,37,38,39,40,41,42,43,44,45,46,47,48,49,50,51,52,53,54,55,56,57,58,59,60,61,62,63,64,65,66,67,68,69,70,71,72,73,74,75,76,77,78,79,80,81,82,83,84,85,86,87,88,89,90,91,92,93,94,95,96,97,98,99,100,101,102,164,165,166]:

- No matter what therapeutic strategy the clinical team chooses, a beneficial practice for treatment and recovery is genetic addiction risk testing and personalized induction of dopamine homeostasis based on genetic test results.
- Indeed, the use of any treatment that reduces stress and enhances resting-state functional connectivity along the brain reward circuitry seems prudent.
- From pre-authorization of very short-term use of opioids to reduce harm to cognitive behavioral therapy, trauma therapy, brain spotting, stress reduction, rsTMS to deep brain stimulation, and 12-stepping, the foundational induction, via epigenetics, of gentle up-regulation of dopaminergic function will be an evidenced basis for the induction of treatment (without complications or side effects) to keep people from any form of dopaminergic dysfunction.
- Genetic addiction risk testing for patients attending a pain clinic provides information about the likelihood of a predisposition for Opioid Use Disorder (OUD) and enables the utilization of less addicting analgesia at the onset of treatment.
- Genetic testing should be the standard of care for all patients attending substance and non-substance (process addictions) dependency programs (i.e., inpatient, outpatient, and intensive outpatient programs).
- Genetic addiction risk testing for early risk identification in children, especially if they have addiction issues in the family (for example, children of alcoholics), combined with precision pro-dopamine regulation prophylaxis, may attenuate or prevent addiction risk.
- Coupling of KB220 precision variants with naltrexone to improve compliance [43].
- Combat cannabis-induced anhedonia in adolescents and adults with RDS using “Precision Behavioral Management” (PBM) to provide precision KB220 formulations [144,146].

## 7. Conclusions

Billions worldwide are affected by SUDs and RDS, which are preventable with PBM, genetic testing for addiction risk, and pro-dopamine precision KB220 formulations selected to treat neurotransmitter deficits. The neurotransmitter deficits are caused by the tested person’s dysfunctional alleles, identified by their genetic test results. This required induction of “dopamine homeostasis” is recommended for frontline tertiary addiction treatment and relapse prevention.

While more in-depth and extensive double-blinded studies combining the GARS test with KB220 precision formulations are encouraged, we believe that this body of evidence presented to support PBM, an important therapeutic and prophylactic modality for the treatment of impaired cognitive control of reward processing as manifested in patients expressing RDS, deserves careful consideration.

## Figures and Tables

**Table 1:** Reward Deficiency Syndrome criteria.

Set 1. Criteria DSM5 Disorders	
A Present or Past Diagnosis or History of These Behavioral Disorders	
Substance Use Process Disorders	Disorders: Alcohol Use Disorder, Opioid Use Disorder, Cannabis Use Disorder, Sedative, Hypnotic, Anxiolytic Use Disorder, Cocaine Use Disorder, Amphetamine Use Disorder, Hallucinogen Use Disorder, Nicotine Use Disorder, Inhalant Use Disorder, Other, Unknown Substance Use Disorder Specifiers: Mild, Moderate, Severe, Early Remission (6–12 months), Sustained Remission (12 + months), in a Controlled Environment, on Maintenance Therapy
Process Disorders	Gambling, Sex, Other Specified Process Disorders
Depressive (and related) Disorders	Major Depression, Dysthymia, Disruptive Mood Dysregulation, SUD/Medication/Medical Condition Inducted Depressive Disorder, Disruptive Premenstrual Dysphoric Disorder
Anxiety Disorders	Generalized Anxiety Disorder, Social Anxiety, Panic Attack Disorder, Separation Anxiety, Selective Mutism, Specific Phobia, SUD/Medication/Medical Condition Inducted Anxiety
Trauma and Stress Disorders	Reactive Attachment, Disinhibited Social Engagement, Post-Traumatic Stress Disorder (PTSD), Acute Stress Disorders
Disruptive, Impulse Control, and Conduct Disorders	Oppositional Defiant Disorder, Intermittent Explosive Disorder, Conduct Disorder, Pyromania, Kleptomania
Personality Disorders	General Personality Disorder, Paranoid Personality Disorder, Schizoid/Schizotypal Personality Disorder, Anti-Social Personality Disorder, Borderline Personality Disorder, Histrionic Personality Disorder, Narcissistic, Personality Disorder, Avoidant Personality Disorder, Dependent Personality Disorder
Obsessive Compulsive Disorders and Related Disorders	Trichotillomania, Excoriation Disorder, SUD/Medical/Medication Inducted OCD Disorder, other Medical Condition, Induced Personality Disorder
Schizophrenic Disorders	Schizophrenia, Schizoaffective Disorder, Schizophreniform Disorder, Delusional disorder, Brief Psychotic Disorder, MH/Medical Catalonia, SUD/Medication/Medical Condition Inducted Psychotic Disorder
Dissociative Disorders	Dissociative Identity Disorder, Dissociative Amnesia, Depersonalization/Derealization Disorder
Other Not Otherwise Specified (NOS) Disorders	Gender Dysphoric Disorder Paraphilic Disorders
Spectrum Disorders	Attention Deficient Disorder, Attention Deficient/Hyperactivity Disorder, Tourette’s Syndrome, Autism
**Set 2. Criteria**	
**Reported history of these symptoms:**	
Novelty seeking	The y trait is associated with exploratory activity in response to novel stimulation, impulsive decision making, extravagance in approach to reward cues, quick loss of temper, and avoidance of frustration.
Impulsivity	The impulsivity construct includes at least two independent components: first, acting without an appropriate amount of deliberation, which may or may not be functional; and second, choosing short-term gains over long-term ones.
Difficulty feeling reward (Anhedonia)	Either a reduced ability to experience pleasure or a diminished interest in engaging in pleasurable activities.
Motivational Anhedonia	A decrease in motivation to participate in pleasurable activities.
Rumination, Obsessive, and Intrusive Negative Thoughts	Possible causes and consequences, as opposed to its solutions.

**Table 2:** Summary GARS test allele function and behavioral risk predisposition [138,139,140,141,142,143,144].

Genetic Variant	Prime Function
G-Allele COMT	Carriers of this allele will have a high activity of synaptic dopamine (DA) reabsorption leading to a reduced interaction at DA receptors.
A-Allele of the DRD1 receptor gene	Carriers of this allele will have a reduced number of DRD1 receptors and lower DA function within the brain reward circuitry. The DRD1 receptor is involved in promoting normal DA function.
A1 variant of the DRD2 receptor gene	Carriers of this allele will have a reduced number of DRD2 receptors up to 40% and, as such, will have a lower DA function within the brain reward circuitry, especially at the Ventral Tegmental Area (VTA) Nucleus Accumbens.
C variant of the DRD3	Carriers of this allele will have a reduced number of DRD3 receptors and have a lower DA function within the brain reward circuitry. Studies have found that this allele associates with risk for Alcohol, Cocaine, and Opioid Use Disorder as well as opioid dependence, especially in the African American population.
C variant of the DRD4 receptor gene	Carriers of this allele will have a reduced number of DRD4 receptors and have a lower DA function within the brain reward circuitry. The DRD4 gene is responsible for normal DA function within the mesolimbic reward cascade, and the C variant is highly associated with risk for ADHD and novelty seeking.
G-Allele of the OPRM1 receptor gene	Carriers of this allele will have a reduced number of Mu opioid receptors. Reduced Mu opioid receptors reduce GABA transmission at the Raphe Nuclei and Substania Nigra, leading to a reduced DA release at the VTA via altered inhibition of the normal Glutaminergic drive.
9 R allele of the DAT1 gene	Carriers of this allele will have a high activity of synaptic dopamine (DA) reabsorption, leading to a reduced interaction at DA receptors.
S or LG allele of the 5-HTTLPR gene	Carriers of these alleles will have a high activity of synaptic serotonin reabsorption, leading to a reduced interaction at serotonin receptors. This paucity leads to a reduced serotonergic transmission at the hypothalamus in the mesolimbic system. The low serotonin activity results in a reduced interaction with the endogenous opioid peptides and, as such, a reduced inhibition at GABA sites.
4 R variant of the MAOA gene	Carriers of this allele will have a high activity of mitochondrial catabolism of both serotonin and dopamine. The high activity will reduce the projection of these neurotransmitters to storage at the pre-neuron vesicles for further release when fired with an action potential.
181 allele of the GABRB3 gene	Carriers of this allele will have an overexpressed GABRB3 that will lead to a higher GABA transmission at the VTA-Glutaminergic site, leading to hypodopaminergia.

## Data Availability

Not applicable.
